# Further Observations and Remarks on the Epidemic Fever, or Influenza

**Published:** 1818

**Authors:** James Carver


### FURTHER OBSERVATIONS AND REMARKS

ON THE

### EPIDEMIC FEVER, OR INFLUENZA,

WHICH STILL EXISTS AND PREVAILS IN AND ABOUT THE

### CITY OF PHILADELPHIA,

UP TO THE PRESENT DATE OF JUNE 15, 1818.

INCLUDING A RECITAL OF FACTS AND CASES OF HORSES BELONGING TO GENTLEMEN NOW RESIDING IN THE CITY OF PHILADELPHIA.

THIS complaint, for a variety of causes which will herein be explained, has now become so very prevalent, as to deserve the attention of the citizens and publick generally. —This distemper, murrain or pest, by many so called, among cattle, at various times, as did some few years ago, prove a terrible scourge to the agricultural interest, and indeed to the world at large. And we learn from history, as I have before stated, that this malignant epidemic, was not unknown among the ancients, which produced such ravages among their horses and their kine as to induce them to make solemn fasts, to appease the Divine wrath, and avert the calamity.

*Epidemic Diseases*—are such as are generally prevalent among all kinds of horses and cattle in general, at some particular time, the liability being the same throughout, and the cause equal; but the origin, apparently dependent on some change taken place in the bodies of all the animals, at that time sponstaneously, or perhaps from something applied from without, as the external air, improper food, or miasmata in any form; these diseases may be contagious or they may not. But in epidemics another induction is now added by some of our first writers on that subject, and that is prevention, to enable him effectually to undertake a preventive treatment, and which is my general plan under all circumstances of disease, whether in the *head*, *the bowels*, *or the feet*, and to do this we must ascertain if possible the exciting cause.

*Endemic Diseases*—by our first writers are considered those peculiar to a particular climate, or place, confining their effects principally to the animals inhabiting those parts; these diseases are thought to be but few in the brute subject.

*Sporadic Diseases*—are those that are confined to one place, and stand in direct opposition to the former, and are a very widely diffused class, and comprehend such as have a peculiar cause, and affect particular constitutions or ages: thus strangles, which generally goes in this country by the name of the distemper, becomes a sporadic disease to young horses, and the distemper a sporadic disease among dogs.

*Specific Diseases*—are such as are peculiar to a particular class of animals: thus *Farcy*, *Glanders*, and *Strangles*, are among the specific diseases of the horse, popularly so called, is one peculiar to dogs.

We shall now treat of the proper knowledge and management of this one and the same disease under different heads: —their *causes*, *symptoms*, *diagnosis*, *prognosis*, and *cure*.

The causes are sometimes involved in obscurity; at others, by attention and observation, they may be discovered; and again, in some instances, they are evident at once. The symptoms of a disease are the immediate effects it produces: Thus an inflamed brain, being productive of vertigo, and delirium, and redness at the eyes, makes delirium and redness of the eyes a symptom of an inflamed brain; but this does not take in any other than the immediate effect; for death is frequently a result of this disease; but death is not a symptom of an inflamed brain.

From the symptoms we form our diagnostic of the disease: that is, we judge of its present state. When we are masters of this, we are led to form a prognosis or opinion of its probable termination.

*The Cure* forms the most important part, and consists in an attempt at assisting Nature in her efforts to produce a natural remission of the disease. If these efforts are wanting or inert, we promote an artificial one, or we attempt to resist the efforts of the disease.

*The malignant epidemic Fever*, called *typhus gravoir*, or *maladie epidemic*, has been raging for some time in the East Indies, though under another form. It has also lately been very severely felt in Europe. And from the accounts which have lately been received from the former as well as the latter place, has still made it doubted whether horses are subject to putrid fever. However, whether they are or not is a discussion we will not now enter into, it being more applicable to our present condensed subject to state that it has carried off some hundreds of horses in both countries; and whoever has observed a horse first attacked with the mild or common epidemic, which then proceeds through all the stages of morbid debility, to throw out the most fœtid sanies, from the nose, the mouth, the stools, and all the secretions betraying the same fœtor; and at last the whole cellular membrane becoming affected with serous effusion, as is now the case with Mr. Charles Watson's mare, which has been seen by Dr. Dorsey and several gentlemen of the city, called and termed by the farriers water farcy, the treatment of which disease being so very different, and the medicines so opposite to what should be applied in such cases that they generally kill; then they say " Oh! the horse was rotten; who the d——l could save him?"

Among horned cattle its ravages are too notorious, and its character too well marked to need argument; and it may too also be considered as a full proof of the liability of the horse to the same, it having occurred that during the ravages of the malignant epidemic among cattle, horses have also become affected, though it must be allowed, that this has but seldom taken place, and on the contrary, in some seasons, there has been undoubted evidence, that horses have fed and housed among kine without being affected. But not only do horses generate and produce this epidemic with a true malignant character in his own person, but that he does now also receive it by the medium of affected cattle, there is also full proof. Many of the French veterinary writers not only describe but treat upon this disease very largely, and among other symptoms of putridity, notice the existence of aphthæ or thrush through the mouth, throat, and alimentary canal. There are also other authors among the Italians and Swiss, who mention the existence of this disease also, as being accompanied by phlegmonous tumours similar to the human anthrax, which does not come to suppuration, but mostly terminate in gangrene.

*The Symptoms.*—Mr. Coleman, professor Blane, and other veterinary writers, say that the malignant epidemic among horses always commences by similar appearances to the mild epidemic. In fact there is every reason to suppose, that it is in many cases only a heightened degree of the common catarrhal affection, pushed into a putrid type by the violence of its action; but at some times we have reason to believe it has raised as a malignant disease only, but fortunately only seldom; but in this kind, in addition to the symptoms accompanying the mild sort, there is purging usually present, and a bloody stinking discharge from the nose and mouth; pulse quick and small; wavering, tottering, and general weakness.

*Treatment.*—When the common epidemic rages with much violence, still more caution ought to be observed with regard to depletion; but the laxitive medicine never omitted; and so soon as the malignancy makes its appearance the most active means must be employed to support strength, and the stable around kept cool. Vinegar fumigation is sometimes good. Green meat of all kinds; but a run at grass will always be attended with the most beneficial effects.

Malt and soft mashes is the best stable food, and sometimes nutritious clysters are advisable; and when the weakness is great, and putridity strong and fast approaching, give port wine and ale gruel, and in case of purging or diarrhœa, starch clysters and drenches, and if the following is given two or three times a day so much the better.

| | |
|---|---|
| Take Sweet spirits of nitre (nitrous æther) | 8 drachms, |
| Mindererius's spirit (liqua ammonia acetatis,) . . . . . | 4 ounces, |
| Camomile infusion or powdered, . | 6 do. |
| Beer yeast, or beer alone, . . | 6 do. |
| Tinct. opii. . . . | 3 drachms. |
| Mix. | |

To this may be added in cases of necessity, two ounces of oak or willow bark.

---

### RECENT ACCOUNT OF CASES IN AND ABOUT THE CITY OF PHILADELPHIA.

The cases in and about the city, from every account that I could collect, have been numerous, and I have this day learnt that it is still prevailing very much about Darby, Chester, and Wilmington. The first first two cases which I was called to were at the paper mills of Lloyd Jones on the old Lancaster road. This gentleman, it appears, had three horses attacked, two old horses and one colt. The first died before I was sent for, and the second before I could get there. This horse, however, was opened by Mr. Jones and myself. The stomach and the intestines were all in a state of inflammation; but the lungs and the heart very much so indeed. Great extravasation of blood had taken place, and from the very high state of inflammation of the pericardium and the liquor surrounding the heart, together with the heart itself, it was evident to me that the disease had terminated in inflamed lungs from ill ventilated stabling.

The third was a promising young colt rising five years old. He was first attacked by great difficulty of swallowing, attended with a dry husky cough, some fever, and swelling under the throat. This colt stood at the south end of a very ill ventilated stable; but to prevent the former occurrence of inflamed lungs, I had him removed to a well ventilated place; and with the usual medicines, and the good nursing, care, and attention of Mr. Jones himself, this horse was saved. I heard of several more in the neighbourhood, which, for want of a proper knowledge of the disease, were carried off by it.

Two others belonging to Mr. George Bumm, of the brick yards; one was a fine stallion. But I was not called in until by neglect the disease had terminated in inflamed lungs. On seeing the horse I pronounced him a dead one; on feeling the pulse, I found it had risen to 140; and though I entertained no hopes, to please the owner I bled him, applied a very strong blister and several issues under and about the jaws. This horse, it appears, had not laid down for several days and nights, which is always the case in this disease. Before I left the stable I ordered him a bed. The horse laid down, and as I told them so it happened, to rise no more; he died in the night. Neither blister nor issues had taken any effect, which convinced me that gangrene and mortification had already taken place before I was called in.

A few days after a chesnut horse belonging to this man was attacked with the same disease. He came to me immediately, and from the very high state of the pulse and other indications of approaching inflammation, I took from him about five quarts of blood, and while pinning up the neck, the horse being very restless, took up sufficient time to admit of the blood's coagulating, which on examination I found so bad as to warrant my calling the man back (for he had already proceeded homewards.) I took away the same quantity, which, with my other usual applications, the horse in less than a fortnight went to work. In this case it was the second depletion that saved him.

Mr. Thomas Rotch, of Walnut-street, had a horse attacked with this complaint, but so very slightly that a small dose of physic, and a few alteratives soon brought the horse round.* This disease had again made its appearance on the Lancaster line; but I was not called in. I however understood that my former system was strictly adhered to by the direction of Mr. J. Tomlinson; and I believe the whole of the horses attacked got pretty well through it.

Mr. Coleman Fisher, of this city, in Chesnut-street, had several horses attacked in his stable in town, as well as on his farm; the whole of which, by his own good care, management, attention, and nursing, he got them all through it. Mr. Fisher, in the whole of these cases, was his own doctor, occasionally consulting me as physician.

Mayor Wharton, of the city of Philadelphia, had two very fine colts attacked with this complaint. They both suffered very much. The running at the nose was very profuse and fœtid; and in the youngest colt was evidently attended with some danger. I was taken very ill at the time, and could not attend, which induced the mayor to pay strict attention to them himself, and by repeatedly consulting me, together with his own good nursing, and strict and regular application of my medicines, which when the horses were in their convalescent state, had joined to them arsenic, which was admi-

* It should be remembered that when ever a horse severely attacked with inflamed lungs once begins to lay down, rises again, and seems refreshed from having done that, it is always the most favourable omen that the disease has taken another turn, and with only common care may be saved; but when a horse who has been attacked some time, and nothing done for him, and on being called in, as in Mr. Bumm's case, and the pulse very high and very strong, once lays down, he never rises again.

nistered with the mayor's own hands, both horses recovered, and are doing well.

---

### CIRCUMSTANCES AND PARTICULARS RELATIVE TO MR. WATSON'S GRAY MARE.

*Being an interesting case, was visited by several gentlemen of this city.*

On or about the 10th of June a celebrated gray mare, seven years old, owned by Mr. C. Watson, of this city, was attacked with an inflammatory anasarcous swelling of the legs, with some cough and a running at the nose. Being requested by Mr. Watson to examine and attend her, I found the mare with her head hanging in the manger, standing wide, and apparently labouring under great debility. The jaws were somewhat swelled, with a running of both nostrils; pulse about 80. Taking all the symptoms into consideration, and from every inquiry I could collect from his groom, I found the mare was attacked with the prevalent complaint called the influenza, though I at first took it for inflamed lungs; but the groom informing me she laid down every night, convinced me it could not be that complaint. The mare's legs were considerably swelled, with various blotches in and about the buttock, and so much so as to cause me for some time to hesitate what the attack really could be. However the mare was labouring under great depression of spirits, refused her food, and laboured very irregularly at her flank, which, together with the state of the pulse, warranted venesection. I took from her five quarts of blood; and as her eyes and nostrils were very much inflamed, I opened both angular veins of the face under the eye, which I also scarified, to relieve the inflammation. I then gave her a strong cathartic, which had the desired effect in keeping her body open for near two days. The pulse, neither from the operation of the physic, nor the quantity of blood taken before, had in the least diminished, which induced me to take away near the same quantity. The swelling in her legs had not abated but rather increased as high up as her abdomen; I accordingly rubbed in a strong blister all over her belly, and under the maxillary jaw, all down the windpipe to the chest, which had little or no effect, which somewhat alarmed me, and caused me to think gangrene had commenced. The following powders, with half an ounce of nitre in her water, was given every night and morning.

℞ Emetic tartar, ℥iij
Arsenic, . . ℥i ss.

Mix and divide into twenty-four doses.

I now thought it advisable to have her turned out, which was accordingly done. She eat grass with great avidity; but the swelling did not subside. I then administered the following draught.

℞ Mindererius's spirit, . . . ℥iiij,
Spirit sweet nitre, . . . . . . ℥ss,
Oxymel squills, . . . . ℥iiij,
Strong decoction of camomile, . . . ℥viij,
Digitalis, . . . . . . ℥ij,

Mixed in some yeast and beer, and gruel for a drink.

The mare was now continued out at pasture, and the powders and nitre continued. The swelling now began to show some symptoms of removal, and settled itself in her head and neck. The nostrils much inflamed and somewhat ulcerated; I therefore rubbed in a strong blister all over her head, jaws, and neck, which had an excellent effect, and in about fifteen days from the commencement of her attack she was returned in a convalescent state to Mr. Watson's stable.

Philadelphia, June 25, 1818. J. C. V. S.

